# Long‐Term Weight Loss in Adults With Overweight or Obesity Using a Breath Biofeedback mHealth App: A One‐Year Follow‐Up of a Randomized Trial

**DOI:** 10.1002/osp4.70106

**Published:** 2025-12-05

**Authors:** Kaja Falkenhain, Dylan A. Lowe, Sean R. Locke, Joel Singer, Ethan J. Weiss, Jonathan P. Little

**Affiliations:** ^1^ School of Health and Exercise Sciences University of British Columbia Okanagan Kelowna British Columbia Canada; ^2^ Pennington Biomedical Research Center Louisiana State University Baton Rouge Louisiana USA; ^3^ Cardiovascular Research Institute, University of California San Francisco San Francisco California USA; ^4^ Department of Kinesiology Brock University St. Catharines Ontario Canada; ^5^ Centre for Health Evaluation and Outcome Sciences, School of Population and Public Health University of British Columbia Vancouver British Columbia Canada

**Keywords:** biofeedback, low‐carbohydrate, mHealth, obesity, weight loss, weight maintenance

## Abstract

**Background:**

Long‐term weight loss success with dietary interventions is notoriously limited. Mobile health (mHealth) interventions offering personalized dietary guidance combined with real‐time biofeedback may enhance long‐term adherence and provide a sustainable solution for weight management.

**Objectives:**

This study reports the prespecified secondary outcome of weight loss at 48 weeks from a parallel‐arm randomized clinical trial (ClinicalTrials.gov: NCT04165707) that aimed to evaluate the long‐term effectiveness and sustainability of a Mediterranean‐style low‐carbohydrate diet delivered via an mHealth application paired with breath biofeedback compared with a calorie‐restricted low‐fat diet application.

**Methods:**

Adults with overweight or obesity (*N* = 155; mean ± SD age, 41 ± 11 years; 71% female; BMI, 33.5 ± 4.7 kg/m^2^) were randomized to either an intervention promoting a Mediterranean‐style low‐carbohydrate diet combined with biofeedback from a handheld breath acetone device or an evidence‐based comparator promoting a calorie‐restricted, low‐fat diet. Participants recorded their daily weights using Bluetooth scales. Weight loss over 48 weeks was analyzed using a linear mixed‐effects model, incorporating all available daily weight measurements from participants who provided at least one follow‐up measurement.

**Results:**

At 48 weeks, participants using the breath biofeedback mHealth app achieved clinically meaningful weight loss (−9.54 kg, 95% CI: −12.27 to −6.81 kg). In contrast, participants using the low‐fat diet app did not achieve statistically significant weight loss (−2.68 kg, 95% CI: −5.49 to 0.14 kg), resulting in a statistically significant between‐group difference (−6.9 kg, 95% CI: −10.8 to −2.9, *p* < 0.001). No adverse effects were reported in either group.

**Conclusions:**

This study demonstrates that a Mediterranean‐style diet promoting carbohydrate restriction coupled with biofeedback support delivered via an mHealth app results in clinically meaningful sustained weight loss at 48 weeks. Given its practicality and demonstrated effectiveness, this approach presents a promising non‐pharmacological alternative or complement for longer‐term weight management.

## 1 Introduction

In the US, 45% of adults attempt weight loss each year [1], but long‐term success remains challenging. Mobile health (mHealth) applications (apps) provide scalable support through the facilitation of self‐monitoring behaviors (e.g., tracking of foods or weight), notifications, and educational content, but evidence supporting long‐term effectiveness is limited.

A potential limitation of traditional dietary mHealth applications is their near‐exclusive focus on self‐reported inputs such as calorie counting or tracking of macronutrients, rather than objective real‐time physiological outcomes [2, 3, 4]. This reliance on detailed self‐monitoring can be burdensome, is notoriously prone to inaccuracies in reporting, and may lead to user fatigue and attrition [5]. Furthermore, users are often instructed to follow a set of dietary rules but receive no immediate, personalized, or physiological feedback on how their adherence is impacting their underlying metabolism [6]. This lack of a tangible reinforcement loop may diminish self‐efficacy and make it difficult to sustain motivation. By contrast, in type 2 diabetes, real‐time continuous glucose monitoring demonstrates how continuous biological feedback can be used as a behavior‐change tool to support self‐management and improve glycemic outcomes [7].

The “Key to Health” intervention (Keyto Inc.) was designed to address this gap by integrating a novel element of personalized physiological biofeedback. The program combines its dietary guidance delivered via an mHealth app with a handheld breath acetone biofeedback device [8]. This device enables users to directly monitor their metabolic response to their dietary choices, providing a personalized index of their adherence and its impact on metabolism, including ketone production and fat oxidation. Breath acetone is a reliable, non‐invasive proxy that reflects whether the body is primarily utilizing fat or carbohydrates for energy [9, 10]. By providing this objective metabolic data in real‐time, the app is designed to shift the focus from the burdensome task of input tracking (i.e., “What did I eat?”) to the real‐time outcome (i.e., “How is my body responding?”). This biofeedback mechanism is hypothesized to be a key driver of success by creating a positive reinforcement loop; users can see in real time the connection between their food choices and their metabolic state, which may enhance dietary adherence, increase engagement, and provide a non‐scale‐based metric of success.

The breath sensor device is paired with an app that provides a specific dietary framework: a low‐carbohydrate Mediterranean‐style eating pattern characterized by reduced consumption of refined carbohydrates. Although low‐carbohydrate and ketogenic diets have gained popularity as weight‐loss strategies that can result in substantial and clinically meaningful weight loss [11, 12], long‐term adherence remains challenging due to the restrictive nature of the diet [13]. The “Key to Health” app provides a more flexible approach that aligns with recommendations from major health organizations to reduce refined carbohydrates and added sugars [14, 15] while simultaneously embracing the principles of the Mediterranean diet. This dietary pattern, characterized by a high intake of vegetables, unsaturated fats, and lean proteins, has demonstrated cardiovascular benefits [16] and weight loss efficacy [17, 18]. Specifically, previous analyses of a randomized controlled trial comparing the “Key to Health” mHealth dietary intervention coupled with the biofeedback device to an evidence‐based calorie‐restricted low‐fat comparator (WW app, WW International Inc.) demonstrated that the former was effective for weight loss and metabolic health improvements at 12 and 24 weeks [17]. The purpose of the present prespecified secondary analysis was to determine whether the observed changes in weight were durable over the long term by evaluating sustained effectiveness over 48 weeks.

## 2 Methods

### 2.1 Design

This analysis evaluated the prespecified secondary outcome of weight loss over 48 weeks from a pragmatic parallel‐arm randomized clinical trial comparing weight loss and metabolic health between a breath biofeedback mHealth app promoting a Mediterranean‐style, low refined‐carbohydrate diet and a calorie‐restricted, low‐fat diet app as an active comparator in adults with overweight or obesity. The primary intervention phase was 12 weeks, with secondary endpoints at 24 and 48 weeks. Trial outcomes assessed at 12 and 24 weeks have been published previously [17, 18], and this analysis reports on weight loss outcomes assessed at 48 weeks. The trial was conducted remotely out of Canada with participating individuals living in California. It was registered on ClinicalTrials.gov (NCT04165707) and the protocol has been published (DERR1‐10.2196/19053) [8]. There were no substantive changes to the trial design, outcomes, or planned analyses after the trial commenced.

### 2.2 Ethics Approval

The study was approved by the University of British Columbia's clinical research ethics board (H19‐01341), and all participants provided digital informed consent prior to data collection.

### 2.3 Participant Recruitment

As described previously [17], participants were recruited between December 1, 2019, and August 11, 2020 from the general community in California, United States, via web‐based advertisement and listservs, completed an eligibility screening questionnaire, and provided digital informed consent. All participants received a Bluetooth scale (iHealth Lina) that automatically transmitted daily weight data to the study team. Participants downloaded the app that corresponded to their randomization assignment (i.e., either the breath biofeedback mHealth diet app or the calorie‐restricted, low‐fat diet app) with a study‐provided login and had a brief onboarding call with study staff before following the dietary intervention based on guidance through the app without further interaction with the study team. The randomization schedule was maintained by a third‐party, password‐protected website using variable permuted block sizes and stratified by sex (male, female) and age (18–40, 41–64 years). No additional concomitant care or interventions were provided to the participants. A detailed CONSORT flow diagram describing participant recruitment, randomization, follow‐up, and analyses has been published previously and is presented in Figure S1 [17].

### 2.4 Mobile Weight Loss Intervention

#### 2.4.1 Intervention Group

The breath biofeedback mHealth diet app (Key to Health) delivered a dietary intervention through educational articles, recipes, and meal plans emphasizing a Mediterranean‐style diet with high‐fiber vegetables, unsaturated fats, and lean protein, while minimizing the intake of refined carbohydrates. Users were not required to track food intake precisely but were advised to follow the recommended portion sizes and eat to satiety. A handheld breath acetone device paired with the app provided real‐time biofeedback displayed as numeric levels (0–6+), serving as a proxy measure of fat metabolism, and guided dietary adjustments (e.g., further reduction in refined carbohydrates) based on the measured level. The app also contained a chat function to allow users to interact with each other.

#### 2.4.2 Comparator Group

The comparator intervention was a calorie‐restricted, low‐fat diet app (WW International Inc.) that required users to track food intake. Using a personalized daily point budget, participants are instructed to log food items which are assigned points based on their caloric, protein, saturated fat, and sugar content. The program also features a list of “zero‐point” foods that can be consumed freely without affecting the daily point allotment. This app also contained educational material, recipes, and support groups for interaction with other users.

### 2.5 Daily Weight Measurements

Participants were instructed to record daily weights using the provided Bluetooth scale, which automatically synchronized the recorded weights with an online platform via which the research team was able to access the data. Baseline weight was defined as the first recorded weight on the trial start date or the closest available measurement. Changes in absolute (kg) and relative (%) weight loss from baseline were calculated for all weight measurements collected from each participant. Due to the nature of the dietary interventions, participants were not blinded to their allocated intervention. However, investigators responsible for data analysis remained blinded to group allocation until analyses were completed.

### 2.6 Statistical Analysis

Summary statistics and baseline descriptives are presented as mean and standard deviation for continuous data, and N and percentage for categorical data. Data on change in weight derived from the Bluetooth scales provided to participants were analyzed using an intention‐to‐treat constrained longitudinal linear mixed‐effects model that incorporated all available daily weight measurements throughout the study period. This approach allowed robust estimation of sustained weight loss across the 48‐week study intervention phase, including all randomized participants who provided at least one follow‐up weight measurement. Furthermore, this model is able to handle missing data resulting from participants missing daily weight recordings or dropping out. Additionally, a sensitivity analysis was conducted to explore whether the weight loss trajectory (i.e., the difference in change in body weight at 24 weeks) differed between those that did versus did not record a body mass measurement within the last 30 days of the intervention period; this analysis was conducted via a linear mixed model that included an interaction between time and a dummy variable representing data availability. Statistical analyses were conducted in R (version 4.3.2) and statistical significance was set at *p* < 0.05. A comprehensive statistical analysis plan including detailed sample size calculations has been published previously [8, 17].

## 3 Results

### 3.1 Participants

Participant baseline characteristics have been previously published [10] and are shown in Table 1.

**TABLE 1:** Baseline characteristics of participants.

	Total	Breath biofeedback mHealth app	Calorie‐restricted, low‐fat diet app
*N*	155	77	78
Age, mean (SD), years	41 (11)	42 (11)	41 (11)
Female, *N* (%)	110 (71)	55 (71)	55 (71)
Male, *N* (%)	45 (29)	22 (29)	23 (29)
Weight, mean (SD), kg	94.4 (16.0)	94.7 (17.1)	94.1 (14.7)
BMI, mean (SD), kg/m^2^	33.5 (4.7)	33.5 (4.7)	33.6 (4.7)
Ethnicity, *N* (%)			
Black	6 (4)	2 (3)	4 (5)
Latin American	23 (15)	12 (16)	11 (14)
Middle Eastern	2 (1)	2 (3)	0 (0)
East Asian	13 (9)	5 (6)	8 (10)
South Asian	5 (3)	3 (4)	2 (3)
Southeast Asian	5 (3)	4 (5)	1 (1)
White	71 (46)	37 (48)	34 (44)
Multiracial/not specified	30 (19)	12 (15)	18 (23)
Education, *N* (%)			
Apprenticeship or trades certificate or diploma	10 (7)	7 (9)	3 (4)
College certificate or diploma	26 (17)	11 (14)	15 (19)
High school graduate	13 (8)	6 (8)	7 (9)
Less than high school	3 (2)	1 (1)	2 (3)
Post‐graduate degree	50 (32)	24 (31)	26 (33)
University certificate, diploma or degree	48 (31)	26 (34)	22 (28)
Not specified	5 (3)	2 (3)	3 (4)
Income, *N* (%)			
$0–$25,000	18 (12)	6 (8)	12 (15)
$25,000–$50,000	18 (12)	9 (12)	9 (12)
$50,000–$75,000	19 (12)	8 (10)	11 (14)
$75,000–$100,000	16 (10)	10 (13)	6 (8)
$100,000–$125,000	18 (12)	10 (13)	8 (10)
$125,000–$150,000	9 (6)	4 (5)	5 (6)
$150,000–$175,000	8 (5)	3 (4)	5 (6)
$175,000 +	28 (18)	15 (19)	13 (17)
Not specified	21 (13)	12 (16)	9 (12)

*Note:* Data presented as mean (SD) or *N* (%) as indicated.

Abbreviations: BMI, body mass index; SD, standard deviation.

### 3.2 Weight Loss

**FIGURE 1:**
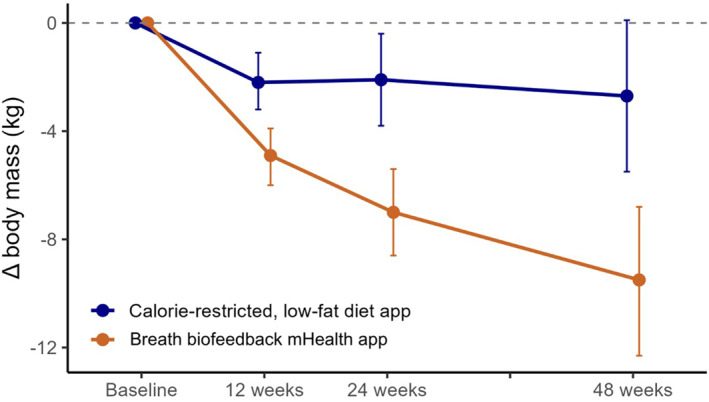
Changes in weight from baseline over time in the calorie‐restricted low‐fat diet app (blue) and the breath biofeedback mHealth app (orange). Shown are effect estimates alongside 95% confidence intervals at 12, 24, and 48 weeks of the intervention.

Weight loss data are shown in Table 2; weight loss data at 12 and 24 weeks have been previously reported [17] and are presented here again for reference. Across the 48‐week intervention phase, a total of 15,767 daily weight entries were recorded by participants on their at‐home Bluetooth scales. Data availability across the intervention period is shown in Table S1, and the CONSORT flow diagram is shown in Figure S1; between intervention weeks 24 and 48, a total of *N* = 45 (58%) and *N* = 35 (45%) individuals recorded at least one body weight in the breath biofeedback mHealth app group and the calorie‐restricted low‐fat group, respectively. At the 48‐week follow‐up time point, participants using the breath biofeedback mHealth app achieved a significant weight loss of −9.54 kg (95% CI: −12.27 to −6.81), whereas participants using the calorie‐restricted low‐fat diet app achieved a weight loss of −2.68 kg (95% CI: −5.49 to 0.14) that was not statistically significant. As a result, the between‐group difference in weight change over time was statistically significant, demonstrating greater weight reduction with the breath biofeedback mHealth app (−6.9 kg, 95% CI: −10.8 to −2.9, *p* < 0.001) (Figure 1). Weight loss expressed as the percent baseline body weight showed similar effects (Table 2). The weight loss trajectory at the 24‐week time point did not differ between those that did and those that did not record a follow‐up body weight measurement within one month of the 48‐week time point (*p* = 0.39), suggesting no differential change in weight between those that did versus did not complete the entire intervention. No adverse effects were reported in either group.

**TABLE 2:** Within‐ and between‐group effect estimates in weight loss.

	Breath biofeedback mHealth app	Calorie‐restricted, low‐fat diet app	Difference between groups	*p* value
Change in body mass at 12 weeks				
kg	−4.9 (−6.0 to −3.9)	−2.2 (−3.2 to −1.1)	−2.8 (−4.2 to −1.3)	< 0.001
%BBW	−5.1 (−6.1 to −4.0)	−2.3 (−3.4 to −1.2)	−2.8 (−4.3 to −1.2)	< 0.001
Change in body mass at 24 weeks				
kg	−7.0 (−8.6 to −5.4)	−2.1 (−3.8 to −0.4)	−4.9 (−7.3 to −2.6)	< 0.001
%BBW	−7.0 (−8.8 to −5.3)	−2.1 (−2.1 to −0.4)	−4.9 (−7.4 to −2.5)	< 0.001
Change in body mass at 48 weeks				
kg	−9.5 (−12.3 to −6.8)	−2.7 (−5.5 to 0.1)	−6.9 (−10.8 to −2.9)	< 0.001
%BBW	−9.5 (−12.2 to −6.8)	−2.6 (−5.4 to 0.1)	−6.9 (−10.7 to −3.0)	< 0.001

*Note:* Data presented as effect estimates alongside 95% confidence intervals derived from a linear mixed effect model including all daily weight measurements recorded by participants on their at‐home Bluetooth scale across the intervention period. %BBW, percent of baseline body weight.

## 4 Discussion

The present study demonstrates that an mHealth app coupled with a breath acetone biofeedback device providing guidance to consume a Mediterranean‐style diet emphasizing low intake of refined carbohydrates may offer a durable and practical solution.

Participants using the breath biofeedback mHealth app were guided to reduce refined carbohydrate intake without needing to strictly adhere to traditional very low‐carbohydrate ketogenic levels. Although they achieved a meaningful carbohydrate reduction compared to the low‐fat diet app group, participants using the breath biofeedback mHealth app reported an average intake of approximately 90 g/day [17], which can be expected to result in average breath acetone levels around 2–3 ppm [17]. These acetone levels are below the traditional nutritional ketosis threshold (4–30 ppm) [9] but notably correspond to levels indicating a caloric deficit even in the context of non‐ketogenic diets [9, 10, 19]. These results suggest that moderate carbohydrate restriction, achieved through a focus on whole foods, high vegetable intake, and low refined carbohydrates, can be effective for weight management without the complexity or dietary restrictiveness commonly associated with traditional ketogenic diets.

The integration of breath acetone biofeedback is a key feature supporting long‐term engagement as it provides tangible, immediate, and actionable feedback on dietary adherence and metabolic status. This mechanism is supported by previous findings from this trial, which found that biofeedback enhances weight loss outcomes by improving dietary adherence, with self‐reported adherence mediating the relationship between biofeedback engagement and weight reduction [18].

The magnitude of weight loss observed at 48 weeks (∼10% of baseline body weight) was clinically significant. Although not a direct head‐to‐head comparison, this outcome is comparable to 1‐year weight loss results reported with semaglutide in both clinical practice and randomized trials, which range widely from approximately 5%–15% body weight [20, 21, 22, 23]. Given the growing interest in GLP‐1 receptor agonists for weight management, our findings highlight the potential for dietary interventions, especially those utilizing biofeedback and promoting flexible, sustainable dietary changes as effective, medication‐free alternatives or complements that may support long‐term behavior modification.

This study has several limitations. First, as a fully remote study, adherence to daily at‐home dietary weight measurements varied among participants. Attrition at the later stages of the study was relatively high in general, and additionally higher in the active comparator group than in the intervention group. Although our sensitivity analyses helped mitigate this limitation by incorporating all available data from the entire study period, caution is warranted in interpreting the results. Additionally, the generalizability of these findings beyond motivated app users remains uncertain. This is particularly of importance given the limited number of people that continued to actively engage with the intervention at the 48‐week time point, which can likely be attributed to multiple factors, including the low‐touch, remote nature of the intervention and the study having been conducted during the COVID‐19 pandemic. Future research is warranted to explore outcomes across broader and more diverse populations, evaluate real‐world effectiveness, and assess the cost‐effectiveness of this intervention.

In conclusion, this study provides promising evidence that a breath biofeedback mHealth app promoting a Mediterranean‐style diet with moderate carbohydrate restriction can deliver clinically meaningful and sustainable weight loss. Given rising obesity prevalence and associated healthcare costs, scalable and pragmatic interventions such as this breath biofeedback diet app have significant potential for broad public health impact.

## Author Contributions

J.P.L. designed the trial with input from E.J.W., J.S., S.R.L., K.F., and D.A.L., K.F. and J.P.L. designed the analyses. K.F., D.A.L., and S.R.L. acquired the data. K.F. performed the statistical analyses with guidance from J.P.L. and J.S., K.F. and J.P.L. drafted the manuscript. J.P.L. had full access to all the data and takes responsibility for the integrity of the data and the accuracy of the data analysis. All authors approved the final version of the manuscript.

## Funding

The study was supported by a Canadian Institutes of Health Research New Investigator Award (MSH‐141980) and a Michael Smith Foundation for Health Research Scholar Award (MSFHR 16890) during the conduct of this trial. This study was funded by a Mitacs Accelerate Grant (IT15608).

## Conflicts of Interest

All conflicts are listed in the Acknowledgments.

## Supporting information


**Figure S1:** CONSORT flow chart. Participant flow through the trial.

**Table S1:** Data availability of participants recording at least one body weight measurement on their at‐home Bluetooth scale across the intervention period.
