# Supplementary material for: Long‐Term Weight Loss in Adults With Overweight or Obesity Using a Breath Biofeedback mHealth App: A One‐Year Follow‐Up of a Randomized Trial
Source: Obes Sci Pract. 2025 Dec 5;11(6):e70106. doi: 10.1002/osp4.70106 (PMC12680900; doi:10.1002/osp4.70106)
Supplement: Supplementary file 1 — Figure S1: CONSORT flow chart. Participant flow through the trial. [file OSP4-11-e70106-s001.docx]

Follow-Up

At 48 Weeks

Discontinued intervention (N=10)

Lost to follow-up for primary outcome (N=37)

Did not log follow-up weight (N=9)

Dropped out (N=28)

Discontinued intervention (N=24)

Lost to follow-up for primary outcome (N=30)

Did not log follow-up weight (N=5)

Dropped out (N=25)

Excluded (N=1380)

Not meeting inclusion criteria (N=1358)

Declined to participate (N=15)

Other reasons (N=7)

Analysis

Randomized (N=155)

Allocation

Supplementary Figure S1. CONSORT flow chart. Participant flow through the trial.

Allocated to calorie-restricted, low-fat diet app (N=78)

Received allocated intervention (N=71)

Did not receive allocated intervention (N=7)

Ineligible after randomization (N=1)

Did not start (N=6)

Allocated to breath biofeedback mHealth app (N=77)

Received allocated intervention (N=75)

Did not receive allocated intervention (N=2)

Ineligible after randomization (N=1)

Did not start (N=1)

Completed 48-week intervention (N=17)
Contributed data between 24-48 weeks (N=35)

Completed 48-week intervention (N=28)
Contributed data between 24-48 weeks (N=45)

Enrolment

Assessed for eligibility (N=1535)
