# Supplementary material for: Long‐Term Weight Loss in Adults With Overweight or Obesity Using a Breath Biofeedback mHealth App: A One‐Year Follow‐Up of a Randomized Trial
Source: Obes Sci Pract. 2025 Dec 5;11(6):e70106. doi: 10.1002/osp4.70106 (PMC12680900; doi:10.1002/osp4.70106)
Supplement: Supplementary file 2 — Table S1: Data availability of participants recording at least one body weight measurement on their at‐home Bluetooth scale across the intervention period. [file OSP4-11-e70106-s002.docx]

**Table S1. Data availability of participants recording at least one body weight measurement on their at-home Bluetooth scale across the intervention period**

|  | Total  (N=155) | Breath biofeedback mHealth app  (N=77) | Calorie-restricted, low-fat diet app  (N=78) |
| --- | --- | --- | --- |
| Month 1 | **145 (94)** | **75 (97)** | **70 (90)** |
| Week 1 | 143 (92) | 73 (95) | 70 (90) |
| Week 2 | 139 (90) | 71 (92) | 68 (87) |
| Week 3 | 133 (86) | 69 (90) | 64 (82) |
| Week 4 | 129 (83) | 66 (86) | 63 (81) |
| Month 2 | **133 (86)** | **68 (88)** | **65 (83)** |
| Week 5 | 124 (80) | 63 (82) | 61 (78) |
| Week 6 | 121 (78) | 62 (81) | 59 (76) |
| Week 7 | 114 (74) | 58 (75) | 56 (72) |
| Week 8 | 106 (68) | 53 (69) | 53 (68) |
| Month 3 | **114 (74)** | **61 (79)** | **53 (68)** |
| Week 9 | 106 (68) | 55 (71) | 51 (65) |
| Week 10 | 95 (61) | 48 (62) | 47 (60) |
| Week 11 | 101 (65) | 52 (68) | 49 (63) |
| Week 12 | 88 (57) | 49 (64) | 39 (50) |
| Month 4 | **99 (64)** | **52 (68)** | **47 (60)** |
| Week 13 | 88 (57) | 46 (60) | 42 (54) |
| Week 14 | 85 (55) | 44 (57) | 41 (53) |
| Week 15 | 70 (45) | 39 (51) | 31 (40) |
| Week 16 | 70 (45) | 40 (52) | 30 (38) |
| Month 5 | **83 (54)** | **44 (57)** | **39 (50)** |
| Week 17 | 67 (43) | 38 (49) | 29 (37) |
| Week 18 | 74 (48) | 42 (55) | 32 (41) |
| Week 19 | 63 (41) | 36 (47) | 27 (35) |
| Week 20 | 62 (40) | 33 (43) | 29 (37) |
| Month 6 | **80 (52)** | **45 (58)** | **35 (45)** |
| Week 21 | 72 (46) | 42 (55) | 30 (38) |
| Week 22 | 68 (44) | 38 (49) | 30 (38) |
| Week 23 | 60 (39) | 35 (45) | 25 (32) |
| Week 24 | 58 (37) | 36 (47) | 22 (28) |
| Month 7 | **73 (47)** | **43 (56)** | **30 (38)** |
| Week 25 | 58 (37) | 36 (47) | 22 (28) |
| Week 26 | 60 (39) | 41 (53) | 19 (24) |
| Week 27 | 55 (35) | 35 (45) | 20 (26) |
| Week 28 | 50 (32) | 29 (38) | 21 (27) |
| Month 8 | **60 (39)** | **35 (45)** | **25 (32)** |
| Week 29 | 48 (31) | 30 (39) | 18 (23) |
| Week 30 | 47 (30) | 30 (39) | 17 (22) |
| Week 31 | 43 (28) | 30 (39) | 13 (17) |
| Week 32 | 46 (30) | 29 (38) | 17 (22) |
| Month 9 | **53 (34)** | **34 (44)** | **19 (24)** |
| Week 33 | 41 (26) | 27 (35) | 14 (18) |
| Week 34 | 41 (26) | 27 (35) | 14 (18) |
| Week 35 | 41 (26) | 28 (36) | 13 (17) |
| Week 36 | 39 (25) | 25 (32) | 14 (18) |
| Month 10 | **47 (30)** | **30 (39)** | **17 (22)** |
| Week 37 | 37 (24) | 22 (29) | 15 (19) |
| Week 38 | 37 (24) | 23 (30) | 14 (18) |
| Week 39 | 37 (24) | 21 (27) | 16 (21) |
| Week 40 | 40 (26) | 25 (32) | 15 (19) |
| Month 11 | **45 (29)** | **28 (36)** | **17 (22)** |
| Week 41 | 36 (23) | 24 (31) | 12 (15) |
| Week 42 | 36 (23) | 20 (26) | 16 (21) |
| Week 43 | 34 (22) | 19 (25) | 15 (19) |
| Week 44 | 33 (21) | 19 (25) | 14 (18) |
| Month 12 | **43 (28)** | **26 (34)** | **17 (22)** |
| Week 45 | 27 (17) | 15 (19) | 12 (15) |
| Week 46 | 31 (20) | 18 (23) | 13 (17) |
| Week 47 | 37 (24) | 22 (29) | 15 (19) |
| Week 38 | 30 (19) | 18 (23) | 12 (15) |

Data presented as N (%).
